# Innate and adaptive immunity contribute to the anti-tumor mechanisms of action of OncoVEX^mGM-CSF^

**DOI:** 10.1186/2051-1426-3-S2-P338

**Published:** 2015-11-04

**Authors:** Keegan Cooke, Karen Fitzgerald, Becky Yang, Petia Mitchell, Juan Estrada, Beltran Pedro, Achim K Moesta

**Affiliations:** 1 Amgen, Thousand Oaks, CA, USA; 2 Amgen Inc., South San Francisco, CA, USA; 3 Amgen Inc., South, CA, USA; 4 Amgen Inc., Thousand Oaks, CA, USA; 5 Amgen, Inc., Thousand Oaks, CA, USA

## Background

Talimogene laherparepvec, an investigational oncolytic immunotherapy, is a modified herpes simplex virus type-1 (HSV-1) designed to selectively replicate in tumors and to initiate a systemic immune response to target cancer cells. Intralesional administration of talimogene laherparepvec is intended to result in oncolysis within injected tumors, with lytic cell destruction promoting the local release of progeny virus and tumor derived antigens. GM-CSF, a product of the viral transgene, is also produced locally such that it can recruit and stimulate antigen presenting cells to further enhance systemic anti-tumor immune response.

## Methods

In a retrospective analysis of a Phase III melanoma trial investigators found that about two-thirds of the lesions injected with talimogene laherparepvec shrank 50% or more. Among uninjected tumors similar responses were seen in one third of lesions of the skin and lymph nodes and about a sixth of uninjected visceral lesions providing an indication that the treatment is triggering systemic immune effect.

To further dissect the mechanism of action underlying innate and adaptive immunity following viral administration, a talimogene laherparepvec expressing murine GM-CSF (OncoVEX^mGM-CSF^) was employed in syngeneic tumor models (A20 and CT26).

## Results

When administered intratumorally (3 doses of 5x10^6 PFU) OncoVEX^mGM-CSF^ induces tumor regressions in the injected lesion, but also results in significant anti-tumor effects in a contralateral (uninjected) lesion and marked size increases in injected tumor draining lymph nodes. Flow cytometric profling, gene expression analysis, and IHC revealed infiltration of T cells, innate effector cells and antigen presenting cells in tumors and draining lymph nodes. Innate immune responses in the injected tumor are characterized by the induction of Interferon response gene signatures and result in the systemic release of proinflammatory cytokines. These effects are, at least in part, dependent on STING activity, with in vitro functional assays supporting direct agonism of this pathway by the virus. Systemic anti-tumor responses appear to be T cell driven: T cells expressed higher levels of activation markers as early as 2 days post-treatment in injected tumors, with evidence of T cell activation in contralateral lesion by day 7. To elucidate anti-viral and anti-tumor T cell responses, ex vivo re-challenge assays were performed using splenocyte- and draining lymph node-derived T cells following treatment with OncoVEX^mGM-CSF^; viral treatment increased systemic anti-tumor immunity and showed induction of anti-viral responses.

## Conclusions

In conclusion, in vitro and *in vivo* observations suggest that intratumoral injection of OncoVEX^mGM-CSF^ activates multiple immune-mediated mechanisms of action, leading to T cell activation and induction of anti-tumor immunity in mice.

